# Work Ability in the Year after Rehabilitation—Results from the RehabNytte Cohort

**DOI:** 10.3390/jcm12237391

**Published:** 2023-11-29

**Authors:** Mari Nilsen Skinnes, Rikke Helene Moe, Thomas Johansen, Peter Solvoll Lyby, Kjersti Dahl, Idun Eid, Tor Christian Fagertun, Andreas Habberstad, Tonje Jossie Johnsen, Ingvild Kjeken, Mari Klokkerud, Anita Dyb Linge, Anne Dorte Lyken, Anders Orpana, Tarja Rajalahti, Ross Wilkie, Till Uhlig, on behalf of the RehabNytte Consortium

**Affiliations:** 1 Center for Treatment of Rheumatic and Musculoskeletal Diseases (REMEDY), Norwegian National Advisory Unit on Rehabilitation in Rheumatology (NKRR), Diakonhjemmet Hospital, Diakonveien 12, 0370 Oslo, Norway; marinilsen.skinnes@diakonsyk.no (M.N.S.); rikkehelene.moe@diakonsyk.no (R.H.M.); ingvild.kjeken@diakonsyk.no (I.K.); 2 Faculty of Medicine, University of Oslo, Sognsvannsveien 9, 0372 Oslo, Norway; 3 Norwegian National Advisory Unit on Occupational Rehabilitation, Haddlandsvegen 20, 3864 Rauland, Norway; thomas.johansen@arbeidoghelse.no; 4 CatoSenteret Rehabilitation Centre, Kvartsveien 2, 1555 Store Brevik, Norway; peter.lyby@catosenteret.no; 5 Avonova Rehabilitation Centre, Kapteinveien 9, 3512 Hønefoss, Norway; kjersti.dahl@avonova.no (K.D.); idun.eid@avonova.no (I.E.); 6 Vikersund Rehabilitation Centre AS, Haaviks vei 25, 3370 Vikersund, Norway; tor.fagertun@vikersundbad.no; 7 The Norwegian Federation of Organizations of Disabled People, Mariboesgate 13, 0183 Oslo, Norway; andreas.habberstad@ffo.no; 8 Hernes Occupational Rehabilitation Centre, Instituttvegen 34, 2410 Hernes, Norway; tonje.jossie@hernesinstitutt.no; 9 Faculty of Health Sciences, Oslo Metropolitan University, Pilestredet 46, 0167 Oslo, Norway; mariklok@oslomet.no; 10 Muritunet, Grandegata 58, 6210 Sylte, Norway; anita.dyb.linge@muritunet.no; 11 Sørlandet Rehabilitation Centre, Ola Garsons vei 1, 4596 Eiken, Norway; anne.dorte.lyken@sorrehab.no; 12 Skogli Health- and Rehabilitation Centre, Fredrik Colletts veg 13, 2614 Lillehammer, Norway; anders.orpana@skogli.no; 13 Red Cross Haugland Rehabilitation Centre, Hauglandsvegen 308, 6968 Flekke, Norway; tarja.kvalheim@rkhr.no; 14 School of Medicine, Keele University, Keele ST5 5BG, Staffordshire, UK; r.wilkie@keele.ac.uk

**Keywords:** work ability, RMDs, rehabilitation, work, multidisciplinary, cohort study

## Abstract

Background: There is limited knowledge regarding the impact of rehabilitation on work ability. The aim of this study was to explore factors associated with work ability 12 months following a multidisciplinary rehabilitation program in a cohort with different diagnoses. Methods: Of 9108 potentially eligible participants for the RehabNytte research project, 3731 were eligible for the present study, and 2649 participants (mean age 48.6 years, 71% female) consented to contribute with work-related data, and were included. Self-perceived work ability was assessed by the Work Ability Score (WAS) (0–10, 10 = best), during the follow-up period using paired *t*-tests and logistic regression to examine associations between demographic and disease-related factors and work ability at 12-month follow-up. Results: The mean baseline WAS for the total cohort was 3.53 (SD 2.97), and increased significantly to 4.59 (SD 3.31) at 12-month follow-up. High work ability (WAS ≥ 8) at 12 months was associated with high self-perceived health at the baseline (OR 3.83, 95% CI 2.45, 5.96), while low work ability was associated with a higher number of comorbidities (OR 0.26, 95% CI 0.11, 0.61), medium pain intensity (OR 0.56, 95% CI 0.38, 0.83) and being married or cohabiting (OR 0.61, 95% CI 0.43, 0.88). There were no significant differences in work ability between participants receiving occupational and standard rehabilitation. Conclusions: Work ability increased significantly over the follow-up period. High work ability at 12-month follow-up was associated with high self-perceived health at baseline, while being married or cohabiting, having higher number of comorbidities, and experiencing medium baseline pain intensity was associated with lower work ability. Rehabilitation interventions targeting these factors may potentially enhance work ability, leading to a positive impact on work participation among people in need of rehabilitation.

## 1. Introduction

A substantial population live with disability caused by chronic disease, which affects their ability to participate in working life [1,2]. Work participation is important at a societal level, with labor market participation supporting local and national economies, and at an individual level with benefits for health, function and quality of life [3,4,5]. Longitudinal observational data are warranted to identify factors facilitating work participation and factors associated with an increased risk of poor work outcomes [6].

Work participation is closely associated with work ability [7,8]. Work ability reflects the balance between a person’s resources, such as physical and mental health and functional abilities, personal competence, values and work demands [9,10,11,12]. Environmental factors also affect work ability [13], including workplace characteristics (context, tools and human relations), the social security and benefit system, and the healthcare system [10,14,15]. 

Assessing work ability necessitates acknowledging its multidimensional nature and diverse measurement purposes [11,15]. In a rehabilitation setting, tools for assessing work ability includes questionnaires measuring health and work variables, interview guides, and physical and cognitive evaluation tests [15]. Self-reported work ability is considered a useful prognostic factor for prolonged sick leave and disability pension [8,16,17], and it is suggested to be more important than clinical factors in cancer survivors with respect to a return to work (RTW) [18].

Rehabilitation can be defined as a “multimodal, person-centered, collaborative process, including interventions targeting a person’s capacity (by addressing body structures, functions, and activities/participation) and/or contextual factors related to performance with the goal of optimizing the functioning of persons with health conditions currently experiencing disability or likely to experience disability, or persons with disability” [19]. Work ability is a factor addressed during the rehabilitation in people of working age and can be assessed in order to guide rehabilitation activities [10,20]. Work is targeted in occupational rehabilitation and is offered upon specific referrals. It is defined as “a timely, goal-oriented, and planned process, in which various stakeholders collaborate to provide the necessary means to empower patients in their effort to achieve optimal functional capacity, coping skills, independence and participation in working life” [21]. 

Due to the increasing demand for access to rehabilitation to improve individuals’ capacity to stay in the workforce [6,22,23], it is important to identify factors associated with altered work ability in order to be able to provide efficient rehabilitation interventions and programs. Previous studies have investigated the effectiveness of specialized occupational rehabilitation programs on work ability and work participation [24,25,26,27], as well as addressing rehabilitation for specific diagnostic groups [28], and found that it improves work ability and work participation [3,29]. More knowledge is needed on what factors are associated with future work ability for their consideration in rehabilitation programs, as long-term improvement is important. 

The aim of the present study was to explore factors associated with work ability 12 months following commencement of a multidisciplinary rehabilitation program in a cohort with different diagnoses. Our research questions were:

(1) What individual and disease-related factors are associated with work ability 12 months following rehabilitation?
(2) Do patients undergoing specific occupational rehabilitation exhibit significant differences in work ability compared to those receiving standard rehabilitation?
(3) What benefit statuses are associated with work ability at the 12-months follow-up?
(4) What line of work is associated with work ability at the 12-months follow-up?

## 2. Materials and Methods

### 2.1. Study Design and Participants

RehabNytte is a large, prospective longitudinal multicenter rehabilitation cohort with a 12-month follow-up period, consisting of three work packages: (1) Patient engagement; (2) work participation; and (3) monitoring quality and outcomes in rehabilitation, with the present study belonging to work package 2. 

In the RehabNytte cohort, seventeen rehabilitation institutions representing all four Norwegian health regions, recruited more than 3700 participants in the time period from January 2019 to March 2020. The 12-month follow-up was completed in June 2021. Data collection was performed at admission and discharge, and at 3, 6 and 12 months after the admission to rehabilitation. Data were collected through a web-based portal which required authentication in accordance with GDPR data-security level 4. 

Participants in RehabNytte were recruited from both urban and rural areas according to the following criteria: Admittance to rehabilitation in one of the participating institutions, aged 18 years or older; have a good understanding of the Norwegian language and access to the internet. Participants reporting a history of head injury documenting impaired cognitive functioning, severe mental illness with inadequate consent competence or current participation in other rehabilitation programs were excluded. Only participants additionally consenting to data collection on work and benefit status, of working age ≤ 67 years, not retired, not on 100% disability benefits, and responding at baseline were included in the analyses in the current study. Because of the large sample size in this study, no sample size calculations were made. 

Admission to rehabilitation was based on referrals by either a specialist physician or a general practitioner. Types of rehabilitation provided by the different multidisciplinary teams were characterized by heterogeneity, with different structures, contents and durations; including both standard rehabilitation where RTW was a secondary goal, and specific occupational rehabilitation where RTW was the main goal. Occupational rehabilitation was offered to sick-listed workers, as well as to people with reduced work ability, which made them unable to access or maintain income-generating work [30]. These peoples’ needs are often more complex because of the nature of their illness, the duration of their absence from work, and their work and home circumstances [31,32].The occupational rehabilitation program contained an assessment process (including an assessment of work ability), cognitive-behavioral approaches, development of an RTW plan, education promoting self-care and pain management, education on activity and work, exercise and communication with the workplace and other stakeholders, as well as an RTW coordinator in the interdisciplinary team [33].The duration of the rehabilitation intervention varied from one week up to 6 months. The intervention consisted of both inpatient and outpatient rehabilitation programs or a combination of these. Common for all rehabilitation institutions were multidisciplinary rehabilitation interventions tailored to individual needs, provided either one-to-one, in a group setting or in combination. The multidisciplinary teams consisted of physiotherapists, occupational therapists, nurses and medical doctors. Some institutions also included other professionals, such as social workers, work consultants, psychologists, sports pedagogues, nutritionists and pharmacists. 

### 2.2. Ethical Considrations

The RehabNytte project was considered by the regional ethical committee as not requiring approval, due to the objective to evaluate the delivery of rehabilitation services (2018/1645/REK Sør-Øst A). The study was recommended by the data protection officer at Diakonhjemmet Hospital (DS-00040), dated 17 October 2018, and registered in ClinicalTrials.gov (NCT03764982). All participants received oral and written information about the study, and signed an informed consent in accordance with the declaration of Helsinki [34].

#### Patient Engagement

To ensure end-user relevancy, highly experienced patient research partners were broadly represented in this research project, and were actively involved with the research team from the very first brainstorming, to developing the project plan, study design and implementing the study. They participated in the interpretation of findings and prepared manuscripts, and will help disseminate the results along with the other authors.

### 2.3. Outcome Measure

The primary outcome was self-perceived work ability, measured by the Work Ability Score (WAS) [35]. The Work Ability Score is considered a valid, brief alternative for the Work Ability Index in determining work ability, and has been shown to correlate strongly with the Work Ability Index [7,36]. It allows patients to compare their current work ability to their lifetime best on a 0–10 scale (10 = best). A change of 1.5 points can be interpreted as a clinically meaningful change [37]. The psychometric measurement properties have been tested and found satisfactory in a rehabilitation setting [38]. 

### 2.4. Explanatory Variables

Work-related factors collected at baseline were: work status (full work, part-time or absent from work); current work situation (income-generating work, sick-listed, disability benefits, work assessment allowance, student, jobseeker, unpaid work, retired or other); and current or last line of work (manager, professional, technician or associate professional, service-, sales- or care-worker, craft and related trades worker or machine operator, elementary occupation, work-training or apprentice, or other). 

Information on sociodemographic factors, as well as health-related factors, was collected at admission to rehabilitation using a self-administered questionnaire. The diagnosis for admittance to the rehabilitation institution was recorded, and participants checked a list of 19 common comorbidities. Smoking status had the response options: yes–daily, yes–sometimes, no–never and no–quit. Marital status had two response options, single or married/cohabiting. To obtain a broader picture of the participants’ demands outside of paid work, one question about care tasks in or outside of the family (yes/no) and one question about having children living at home (yes/no) were included. The highest completed education had the response options; primary school (≤10 years), secondary school (≤12 years), or upper vocational education or university (>12 years). The geographic region was based on the location of the rehabilitation institutions.

The current pain status (yes/no) was reported at admission to rehabilitation, along with a question regarding pain intensity during the past week, using a numeric rating scale (NRS): 0 equals no pain, and 10 is the worst imaginable pain. Self-reported health was reported as perceived health using the visual analogue scale (VAS) of the EQ-5D-5L questionnaire (EQ VAS), where 0 indicates the worst imaginable health, and 100 indicates the best imaginable health [39]. The EQ-5D has satisfactory psychometric properties in Norwegian rehabilitation settings and in different diagnostic populations, including patients with rheumatic and musculoskeletal diseases [40,41]. 

### 2.5. Statistical Analysis

Descriptive statistics were used for the sample population with percentages, and the mean or median applied as applicable. Groups were compared using either *t*-tests and ANOVA or the Chi-squared test. Paired *t*-tests were used to examine changes in WAS between baseline and follow-up timepoints for the total population, as well as for the standard rehabilitation and occupational rehabilitation groups, respectively. Independent samples the *t*-test were used to explore differences in work ability in the standard and occupational rehabilitation group.

Bivariate logistic regression was conducted to examine the association between explanatory variables and WAS at the 12-months follow-up as the dependent variable. A priori, the set of explanatory variables included age, gender, diagnosis, comorbidities, education, body mass index, smoking status, geographic region, pain intensity, self-reported health, caregiving, children living at home and marital status [15]. These variables were included in a partly adjusted multivariable model for work ability with the inclusion criteria set at *p* < 0.20 [42], while adjusting for age, gender, diagnosis, comorbidities, geographic region, and baseline work ability, and further included in new multivariable models using backwards stepwise regression, still adjusting for the same explanatory variables. The final model retained statistically significant explanatory variables at the *p* < 0.05 level. Adjusted odds ratios (OR) and 95% confidence intervals (CI) were calculated to describe associations with WAS in the multivariable model. For the subgroup analysis, the variables benefit status and main line of work were added individually to the final logistic regression model for work ability at 12-months follow-up.

For comparison of work ability in the responder and non-responder groups, the work ability responders were defined as participants responding to WAS at all timepoints, whereas non-responders included those who did not complete the WAS across all timepoints. For the logistic regression analysis, WAS was grouped into low work ability ≤ 7 and high work ability ≥ 8. This categorization combined the suggested categories of poor (0–5 points) with moderate (6–7 points), and good (8–9 points) with excellent (10 points) [11]. Main line of work was grouped into four categories: manager and professional; technician or associate professional; service, sales and care-workers, craft and related trades of workers or machine operators; work training or apprenticeship; elementary occupations; and other. Age was grouped into the following categories: 18–39, 40–49, 50–59 and 60–66 years. The main diagnosis was grouped into three main categories: rheumatic and musculoskeletal diseases (RMDs), cancers and other, where the other category consisted of smaller groups of different diagnoses such as neurological, cardiological and pulmonary diseases and lifestyle-related conditions such as diabetes or obesity (Appendix B, Table A3). The number of comorbidities were summarized and grouped into four categories: none, 1–2, 3–4 and 5 or more. Height and weight were used to calculate body mass index (BMI), and grouped into the following categories: underweight (<18.5), healthy ≥18.5–<25), overweight (≥25–<30) and obesity (≥30) [43]. Tobacco use was grouped into never, sporadic and daily. Geographic region was grouped into three of the four main health regions of Norway (South-East, West, and Mid and North) where North and Mid were combined because of fewer participating rehabilitation institutions in these two regions. Education level was grouped to 12 years or less, or more than 12 years of education. The NRS pain scale was grouped into tertiles based on participants’ distribution, with the groups being: 0–5, 6–7 and 8–10. The self-reported health (EQ VAS) was also grouped into tertiles based on participants’ distribution: 0–39 mm, 40–55 mm and 56–100 mm. 

No adjustments were made for missing data. All analyses were conducted using STATA software version 17 [44].

## 3. Results

**Figure 1:**
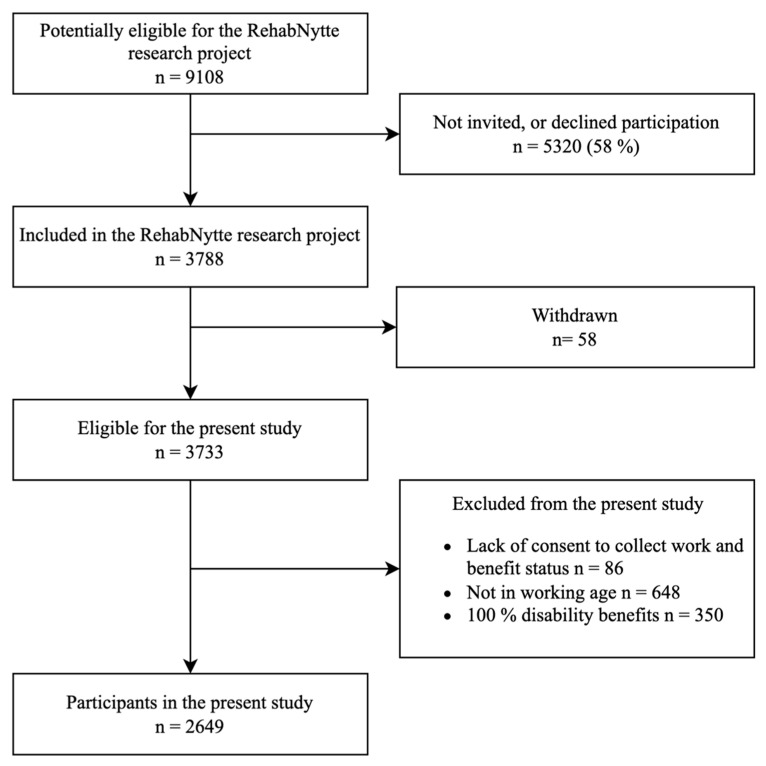
Participant flowchart.

Of the 3788 participants who consented to participate in the study, 86 were excluded because of not consenting to the collection of data on work and benefit status, 648 were not of working age, and 350 received 100% disability benefits. Seventy-one percent (n = 2649) of the included participants were eligible for the analyses and formed the study sample (Figure 1). 

### 3.1. Characteristics of the Study Population

The mean age at admission was 48.6 (SD = 10.9) years. Seventy-one percent (n = 1880) were female, and 42% (n = 1110) were diagnosed with rheumatic and musculoskeletal diseases. Eighty percent of participants reported pain at baseline, with a mean (SD) pain intensity of 5.6 (1.9) (NRS 0-10), and self-reported health (EQ VAS 0-100) of 48 (19) (Table 1). Comparing responders with non-responders, the primary observation was that these groups did not differ substantially. However, the responders were predominantly female, slightly older, had more comorbidities, and had more caring tasks, compared to the non-responders (Table 1).

#### Work-Related Factors

Of the total sample, more than 20% (n = 526) of participants at the baseline reported that their current work situation involved income-generating work, whereas nearly 70% reported receiving various types of health-related benefits (Table 2). More than 70% reported being fully or partly employed before undergoing rehabilitation (Table 2). The responder and non-responder group were similar on work-related factors, except from amount of work prior to rehabilitation, where the responder group were working more part-time and non-responder group were working more full-time. 

### 3.2. Change in Work Ability

The work ability score for all participants increased significantly from baseline mean (SD) of 3.53 (2.97) to 4.59 (3.31) at 12 months, with statistically significant improvements at all timepoints (Table 3). The work ability score increased significantly during the follow-up period for participants receiving standard rehabilitation and occupational rehabilitation, with the largest increase occurring at 12 months for both groups, but without statistically significant differences between the two groups. The occupational rehabilitation group had a significantly lower work ability at the baseline, compared to the standard rehabilitation group. They also had a smaller mean difference at 12 months compared to the standard rehabilitation group, but this was not statistically significant (Table 3).

### 3.3. Predicition of Work Ability Score at 12-Months Follow-Up

High work ability at 12-month follow-up was associated with high self-perceived health at baseline (OR 3.83, 95% CI 2.45, 5.96), as well as being in the 50–59 years age group (OR 2.27, 95% CI 1.36, 3.78) (Table 4). Conversely, participants with cancer (OR 0.27, 95% CI 0.16, 0.47), and participants with a higher number of comorbidities at baseline (OR 0.26, 95% CI 0.11, 0.61) had a reduced chance of high work ability at 12-months follow-up, as did participants reporting medium pain intensity in the last week before admission (OR 0.56, 95% CI 0.38, 0.83) and married or cohabiting participants (OR 0.61, 95% CI 0.43, 0.88) (Table 4). In the final model, education and smoking status lost their statistical significance, and were removed. Twenty-two percent of the variance of work ability was explained by the variables included in the final regression model (R^2^).

#### Subgroup Analyses

When adding benefit status to the final regression model in Table 4, being on sick leave was associated with high work ability, as was age group 50–59 years, and having a medium or high self-perceived health (Appendix A, Table A1). 

When using participants’ current or last main line of work in the final regression model, none of the occupations were associated with high work ability (Appendix A, Table A2). Working as a service-, sale- or care-worker; craft and related trades worker; or machine operator prior to rehabilitation, was associated with lower work ability, as were participants having cancer, increasing number of comorbidities, medium pain intensity and being married or cohabiting. Being in the age group of 50–59 years and reporting a medium or high self-perceived health remained associated with high work ability (Appendix A, Table A2). 

## 4. Discussion

The aims of this study were to identify the extent of changes in, and associations with, work ability at 12 months following a rehabilitation program. Findings indicate a consistent increase in self-perceived work ability during the 12-month follow-up period. High work ability at 12-month follow-up was associated with a high self-perceived health at the baseline, while being married or cohabiting, having a higher number of comorbidities and experiencing medium pain intensity at baseline was associated with lower work ability. There were no differences in work ability between participants receiving standard rehabilitation and participants receiving occupational rehabilitation. 

Work ability increased gradually and significantly during the follow-up period for the cohort as a whole, suggesting that work ability continues to improve following a rehabilitation program. The average increase from baseline to 12-months follow-up approaches the suggested clinically meaningful change of 1.5 points [37]. Others have found that work ability increased both for participants in an occupational rehabilitation program as well as for the control group of sick-listed workers not receiving rehabilitation; however, the increase was larger in the occupational rehabilitation group [24]. Another study comparing the inpatient and outpatient occupational rehabilitation found that work ability increased during the one-year follow-up for both groups [25]. Corroborating these findings is not necessarily expected for participants with chronic or long-term diseases. It has been claimed that an improvement in work ability may be an unrealistic goal in rehabilitation for middle-aged people with chronic conditions whose ability to work has started to decline [45]. 

Being married or cohabiting was associated with lower chances of high work ability one year after rehabilitation, suggesting that living with a partner may be of disadvantage for work ability. This is somewhat unexpected, as it contrasts with other studies that have reported a higher probability of good or excellent work ability among both married or cohabiting participants, whether they are unemployed or working [11,46,47]. One possible explanation could be that people living with a partner already allocate energy and capacity in their lives, to their partners and families. This prioritization could diminish the resources available for work-related activities and negatively affect work ability. This explanation can be supported by studies investigating gender differences, which have found that women often bear a larger burden of unpaid work in the home. This unequal distribution of domestic responsibilities can impact women’s available time and energy, consequently affecting their overall health [48]. This could also have significant economic implications for peoples’ households when they are not engaged in paid work [49]. It is important to note that our analysis is controlled for gender, removing the impact of this factor on the observed association. 

Higher self-perceived health, more comorbidities and higher pain intensity at the beginning of rehabilitation were all associated with work ability one year later, underscoring their significance for self-perceived work ability. These findings align with existing research, reporting associations between self-perceived health and work ability [7,50], and pain and work ability [51], and that having rheumatic and musculoskeletal diseases concurrent with comorbidities is associated with poorer health, higher ratings of pain, psychological distress and work disruptions [22]. People who experience impaired health consider themselves not capable of work, and exhibit a low motivation to participate in the labor market [52]. Possessing a certain level of health is an important factor in the work ability concept [10,53]. The self-perceived health of the participants in the present study was lower than in the Norwegian population norm (mean 77.9 (SD 18.3)), both before and after rehabilitation [54], indicating that the current sample was largely affected by their health problems, which in turn may affect their ability to work.

Participants in the standard rehabilitation group and the occupational rehabilitation group demonstrated a similar improvement in work ability. It is crucial to note that these groups were not subject to randomization; rather, persons referred to occupational rehabilitation programs exhibit specific work-related needs, are usually on health-related benefits for an extended period prior to rehabilitation, and are often referred to occupational rehabilitation late in the process of clarifying work ability [55,56]. Their motivation for RTW may therefore be drained after a long process of clarifying their work capacity, and disability benefits can be considered as a solution to end this process. It is important to underline that employment constitutes a pivotal component of rehabilitation interventions for persons within the working-age group, irrespective of the particular rehabilitation program applied [5,20]. This suggests that the standard rehabilitation group likely addressed work-related factors and work ability within the rehabilitation process as well, albeit without the specialized focus of the occupational rehabilitation program. To enhance the effect of occupational rehabilitation, people in need for such rehabilitation should be referred to earlier in the RTW process.

Work ability is a specific goal in occupational rehabilitation programs. The results of this study have shown that work ability can be improved in a mixed population where the majority received standard rehabilitation. Clinical practice could learn from work-related rehabilitation where there is a systematic effort on improving work ability, drawing on the concept of employability which focuses on activities during rehabilitation, making the person more ready for work [20]. Not all persons in rehabilitation are ready for work, and clinical experience can guide for whom relevant and realistic work-related goals are appropriate. Activities during rehabilitation that focus on employability will cover individual factors such as attitudes, beliefs, coping mechanisms related to work, health and meaning of work as well as contextual and workplace factors in collaboration with relevant stakeholders during rehabilitation and follow-up [20,57]. 

The main strengths of this study were the large sample size, the follow-up period of one year, and a nationwide setting of rehabilitation provided in clinical practice. A limitation is that there are only data on work and benefit status at baseline and not during the follow-up period. Thus, we do not know whether an improved self-reported work ability leads to increased work participation. A lack of specific information with details on the rehabilitation interventions at each institution is another limitation. Objective test results in addition to patient-reported outcomes would have been useful, as well as more data granularity on work and benefits. The observational nature of this study without a control group precludes assumptions on causality. Dichotomizing work ability into high and low removes some of the nuances in work ability, as it is not expected that all participants in this study will achieve high work ability. The grouping of rehabilitation diagnoses allows for heterogeneity, particularly in the diagnostic group ‘other’, which consists of several smaller diagnostic groups, including a mixture of chronic, progressive and non-progressive diseases in the same category. Nevertheless, this reflects the diversity of the population of people receiving rehabilitation. Potentially eligible patients were referred to the rehabilitation institutions in the project period. However, we only have data on those who fulfilled the inclusion criteria and signed an informed consent. The explained variance of the final regression model was at 22%, indicating unmeasured as well as unexplained confounding. In this study, we have only examined a few factors related to work ability. Work ability is a complex concept encompassing numerous factors not included in our model, such as mental health, cognitive functions, personal competence, work demands, and workplace characteristics [10,13,14,15]. 

Future research could involve the use of a register-based control group comprising persons who are not participating in rehabilitation. This provides the opportunity to assess whether rehabilitation is more effective with regards to increasing work ability and work participation, as well as the agreement between work ability, actual work and benefit status; in turn, this may help inform future policy implications. Future research could explore the association between being married or cohabiting and low work ability further, allowing for a better understanding of the mechanisms and factors influencing this relationship. 

In conclusion, we demonstrate a gradual improvement in self-perceived work ability over the 12-month follow-up period. High work ability at 12-month follow-up was associated with a high self-perceived health at the baseline. Being married or cohabiting, having a higher number of comorbidities, and experiencing medium pain intensity at baseline was associated with lower chances of high work ability. There were no significant differences in work ability between participants receiving standard rehabilitation and participants receiving occupational rehabilitation. A comprehensive understanding of the demographic, disease- and work-related factors associated with work ability may better help rehabilitation professionals identify persons or groups with a chance of good and poor work outcomes. The results suggest that targeting self-perceived health, as well as pain management through rehabilitation interventions, may enhance work ability, leading to a positive impact on work participation among people in need of rehabilitation.

## Figures and Tables

**Table 1:** Baseline characteristics for all participants, and comparisons between participants responding and not responding to work ability score at all timepoints.

	All (n = 2649)	WAS Non-Responder (n = 2006)	WAS Responder (n = 643)
Age, years, mean (SD)	48.6 (10.9)	48.2 (11.1)	49.7 (10.4) **
Gender, female n (%)	1880 (71.0)	1380 (68.8)	500 (77.8) **
Diagnosis, n (%)			
Other	927 (35.0)	707 (35.2)	220 (34.2)
Musculoskeletal and rheumatic diseases	1110 (41.9)	844 (42.1)	266 (41.4)
Cancer	612 (23.1)	455 (22.7)	157 (24.4)
Comorbidities, n (%)			
None	635 (23.9)	589 (29.4)	46 (7.2) **
1–2	1255 (47.4)	908 (45.3)	347 (54.0) **
3–4	584 (22.1)	390 (19.4)	194 (30.2) **
5 or more	175 (6.6)	119 (5.9)	56 (8.7) **
BMI, kg/m^2^, median (25 and 75 percentile)	28.4 (24.6, 33.6)	Mean (SD) 29.8 (7.2)	Mean (SD) 30.3 (7.1)
Smokers, n (%)			
Never	974 (43.8)	687 (43.5)	287 (44.6)
Previous/sporadic	1050 (47.3)	738 (46.7)	312 (48.5)
Daily	198 (8.9)	154 (9.8)	44 (6.8)
Married/cohabiting, n (%)	1349 (60.7)	957 (60.5)	392 (61.0)
Caring tasks, yes n (%)	990 (44.6)	701 (44.4)	289 (45.0)
Children living at home, n (%)	965 (43.4)	705 (44.6)	260 (40.4)
Geographic region, n (%)			
West	380 (14.4)	261 (13.0)	119 (18.5) **
South-East	1996 (75.4)	1559 (77.7)	437 (68.0) **
North and Mid-Norway	273 (20.3)	186 (9.3)	87 (13.5) **
High education level > 12 years, n (%)	1046 (47.1)	727 (46.1)	319 (49.7)
Pain in the last week, yes n (%)	1779 (80.2)	1272 (71.5)	507 (28.5)
Pain intensity (0–10), mean (SD)	5.6 (1.9)	5.6 (1.96)	5.5 (1.8)
EQ-5D VAS (0–100), mean (SD)	48.1 (19.1)	48.2 (19.5)	47.7 (18.3)

SD: Standard deviation. EQ-5D: EuroQol 5 dimensions. VAS: Visual analogue scale (100 = best). ** *p* < 0.001.

**Table 2:** Work-related factors at baseline, and comparisons between participants responding, and not responding to work ability score at all timepoints.

	All (n = 2649)	WAS Non-Responder (n = 2006)	WAS Responder (n = 643)
Present work situation, n (%)			
Income-generating work	526 (23.7)	373 (23.7)	153 (23.8)
Sick leave	653 (29.4)	463 (29.4)	190 (29.6)
Disability benefits ^#^	225 (10.1)	152 (9.6)	73 (11.4)
Work assessment allowance	671 (30.2)	478 (30.3)	193 (30.0)
Student	33 (1.5)	29 (1.8)	4 (0.6)
Job seeker	38 (1.7)	26 (1.7)	12 (1.9)
Unpaid work	9 (0.4)	5 (0.3)	4 (0.6)
Other	64 (2.9)	50 (3.2)	14 (2.2)
Work Ability Score (0–10), mean (SD)	3.5 (3.0)	3.6 (3.0)	3.4 (2.9)
Current or last main line of work, n (%)			
Manager	260 (11.8)	181 (11.6)	79 (12.3)
Professional	179 (8.1)	116 (7.4)	63 (9.8)
Technician or associate professional	453 (20.6)	327 (21.0)	126 (20.0)
Service-, sales- or care-worker	392 (14.8)	278 (17.8)	114 (17.8)
Craft and related trades worker or machine operator	416 (18.9)	301 (19.3)	115 (18.0)
Elementary occupations	111 (5.1)	79 (5.1)	32 (5.0)
Work training or apprentice	34 (1.6)	26 (1.7)	8 (1.3)
Other	354 (16.1)	251 (16.0)	103 (16.1)
Amount of work prior to rehabilitation, n (%)			
Full work	976 (44.1)	713 (45.4)	263 (40.9) **
Part-time work	682 (30.8)	453 (28.9)	229 (35.6) **
Not working	554 (25.1)	403 (25.7)	151 (23.5) **

WAS: Work ability score. ^#^ Excluded those on 100% disability benefits. ** *p* < 0.01.

**Table 3:** Change in work ability score from baseline to 12 months for all participants and according to type of rehabilitation.

	Total			Standard Rehabilitation			Occupational Rehabilitation Program		
	Mean (SD)	Paired *t*-Test		Mean (SD)	Paired *t*-Test		Mean (SD)	Paired *t*-Test	
Timepoint, n		Mean Difference (SD)	95% CI of the Difference		Mean Difference (SD)	95% CI of the Difference		Mean Difference (SD)	95% CI of the Difference
Baseline, n = 2211	3.53 (2.97)			3.61 (3.10) ^#^			3.27 (2.49)		
Discharge, n = 1071	3.96 (3.02)	0.42 (1.92) **	0.30, 0.53	4.0 (3.15)	0.43 (1.97) **	0.29, 0.56	3.82 (2.56)	0.39 (1.77) **	0.17, 0.61
3 months, n = 1672	4.22 (3.13)	0.66 (2.22) **	0.55, 0.78	4.26 (3.24)	0.66 (2.23) **	0.54, 0.78	4.06 (2.69)	0.65 (2.20) **	0.42, 0.89
6 months, n = 1464	4.35 (3.20)	0.84 (2.56) **	0.71, 0.98	4.44 (3.28)	0.87 (2.57) **	0.73, 1.02	4.00 (2.87)	0.74 (2.50) **	0.45, 1.02
12 months, n = 1447	4.59 (3.31)	1.0 (2.75) **	0.86, 1.14	4.67 (3.38)	1.02 (2.74) **	0.86, 1.18	4.30 (3.03)	0.93 (2.79) **	0.61, 1.24

SD: Standard deviation. CI: Confidence interval. ** *p* < 0.001. ^#^ Difference at *p* < 0.05 between mean work ability in the standard rehabilitation group and the occupational rehabilitation group at baseline.

**Table 4:** Associations between demographic and disease variables, and high work ability score (≥8) 12 months after rehabilitation using logistic regression analyses. Final model: n = 1125.

	Unadjusted Model		Partly Adjusted Model ^¤^		Final Model ^¤¤^	
Independent variable	OR (95% CI)	*p*-value	OR (95% CI)	*p*-value	OR (95% CI)	*p*-value
Age						
18–39 years ^a^	1.0		1.0		1.0	
40–49 years	1.24 (0.86, 1.79)	0.24	1.21 (0.78, 1.85)	0.39	1.59 (0.92, 2.74)	0.10
50–59 years	1.36 (0.97, 1.90)	0.08	1.56 (1.04, 2.33)	**0.03**	2.27 (1.36, 3.78)	**0.002**
60–66 years	1.30 (0.88, 1.92)	0.20	1.63 (1.01, 2.68)	**0.04**	1.80 (0.97, 3.37)	0.06
Diagnosis						
Other ^a^	1.0		1.0		1.0	
Rheumatic and musculoskeletal diseases	0.56 (0.43, 0.72)	**<0.001**	0.83 (0.60, 1.15)	0.26	1.10 (0.73, 1.67)	0.64
Cancer	0.50 (0.37, 0.67)	**<0.001**	0.44 (0.31, 0.65)	**<0.001**	0.27 (0.16, 0.47)	**<0.001**
Comorbidities						
None ^a^	1.0		1.0		1.0	
1–2	0.97 (0.73, 1.29)	0.84	0.51 (0.33, 0.78)	**0.02**	0.49 (0.27, 0.87)	**0.02**
3–4	0.77 (0.55, 1.08)	0.14	0.43 (0.27, 0.69)	**<0.001**	0.41 (0.22, 0.76)	**0.005**
5 or more	0.35 (0.18, 0.67)	**0.001**	0.21 (0.10, 0.44)	**<0.001**	0.26 (0.11, 0.61)	**0.001**
Education						
≤12 years ^a^	1.0		1.0			
>12 years	1.44 (1.13, 1.83)	**0.003**	1.44 (1.09, 1.90)	**0.01**		
Pain intensity						
0–5 ^a^	1.0		1.0		1.0	
6–7	0.50 (0.36, 0.69)	**<0.001**	0.50 (0.34, 0.72)	**<0.001**	0.56 (0.38, 0.83)	**0.004**
8–10	0.44 (0.28, 0.70)	**<0.001**	0.54 (0.33, 0.89)	**0.02**	0.72 (0.42, 1.23)	0.23
EQ-5D VAS						
0–39 ^a^	1.0		1.0		1.0	
40–55	1.96 (1.37, 2.80)	**<0.001**	2.13 (1.46 3.13)	**<0.001**	2.01 (1.29, 3.12)	**0.002**
56–100	4.97 (3.56, 6.93)	**<0.001**	3.44 (2.36, 5.02)	**<0.001**	3.83 (2.45, 5.96)	**<0.001**
Marital status						
Single ^a^	1.0		1.0		1.0	
Married/cohabiting	0.84 (0.66, 1.07)	0.15	0.65 (0.49, 0.87)	**0.004**	0.61 (0.43, 0.88)	**0.009**
Smoking						
Never ^a^	1.0		1.0			
Previous/sporadic	0.95 (0.74, 1.21)	0.67	0.97 (0.74, 1.29)	0.86		
Daily	0.68 (0.41, 1.14)	0.15	0.75 (0.42, 1.35)	0.34		
Caring tasks						
No ^a^	1.0					
Yes	0.86 (0.67, 1.09)	0.21				

Work ability score (WAS) as the dependent variable, dichotomized into low and high. CI: confidence interval. OR: Odds ratio. EQ-5D: EuroQol 5 dimensions. VAS: Visual analogue scale (100 = best). ^¤^ Individual associations with WAS, adjusted for age, gender, region, diagnosis, comorbidities and work ability at baseline. ^¤¤^ OR adjusted for age, gender, region, diagnosis, comorbidities and work ability at baseline. ^a^ Reference category. Bold indicates *p* < 0.05.

## Data Availability

The data that support the findings of this study are available from the authors upon reasonable request and with permission of the research board.

## Appendix A

**Table A1:** Subgroup analysis with benefit status added to the final logistic regression model for work ability 12 months after rehabilitation, n = 834.

	OR (95% CI)	*p*-Value
Independent variable		
Age		
18–39 years ^a^	1.0	
40–49 years	1.10 (0.48, 2.52)	0.82
50–59 years	3.04 (1.44, 6.40)	**0.003**
60–66 years	1.65 (0.67, 4.08)	0.28
Diagnosis		
Other ^a^	1.0	
Rheumatic and musculoskeletal diseases	1.04 (0.55, 1.96)	0.90
Cancer	0.40 (0.18, 0.88)	**0.02**
Comorbidities		
None ^a^	1.0	
1–2	0.74 (0.29, 1.86)	0.52
3–4	0.77 (0.29, 1.99)	0.58
5 or more	0.42 (0.12, 1.45)	0.17
Pain intensity		
0–5 ^a^	1.0	
6–7	0.54 (0.31, 0.95)	**0.03**
8–10	0.78 (0.39, 1.59)	0.50
EQ-5D VAS		
0–39 ^a^	1.0	
40–55	2.96 (1.61, 5.43)	**<0.001**
56–100	4.17 (2.15, 8.09)	**<0.001**
Marital status		
Single ^a^	1.0	
Married/cohabiting	0.64 (0.39, 1.07)	0.09
Benefit status		
Disability pension ^a^	1.0	
Work assessment allowance	1.40 (0.49, 3.96)	0.53
Sick leave	9.95 (3.90, 25.39)	**<0.001**

Work ability score (WAS) as dependent variable, dichotomized into low/moderate and good/excellent. CI: confidence interval. OR: Odds ratio. EQ-5D: EuroQol 5 dimensions. VAS: Visual analogue scale (100 = best). OR adjusted for age, gender, region, diagnosis, comorbidities and baseline work ability. ^a^ Reference category. Bold indicates *p* < 0.05. Pseudo R^2^ = 0.23.

**Table A2:** Subgroup analysis with line of work added to the final logistic regression model for work ability 12 months after rehabilitation, n = 1118.

	OR (95% CI)	*p*-Value
Independent variable		
Age		
18–39 years ^a^	1.0	
40–49 years	1.49 (0.86, 2.57)	0.16
50–59 years	2.18 (1.31, 3.65)	**0.003**
60–66 years	1.74 (0.93, 3.26)	0.08
Diagnosis		
Other ^a^	1.0	
Rheumatic and musculoskeletal diseases	1.11 (0.73, 1.67)	0.62
Cancer	0.26 (0.15, 0.56)	**<0.001**
Comorbidities		
None ^a^	1.0	
1–2	0.49 (0.27, 0.89)	**0.02**
3–4	0.40 (0.22, 0.75)	**0.004**
5 or more	0.25 (0.11, 0.60)	**0.002**
Pain intensity		
0–5 ^a^	1.0	
6–7	0.56 (0.38, 0.83)	**0.004**
8–10	0.72 (0.42, 1.23)	0.23
EQ-5D VAS		
0–39 ^a^	1.0	
40–55	2.01 (1.29, 3.14)	**0.002**
56–100	3.72 (2.38, 5.82)	**<0.001**
Marital status		
Single ^a^	1.0	
Married/cohabiting	0.59 (0.41, 0.86)	**0.005**
Main line of work		
Manager and professional ^a^	1.0	
Technician or associate professional	0.93 (0.56, 1.57)	0.79
Service-, sales- or care-worker; craft and related trades worker; or machine operator	0.59 (0.37, 0.93)	**0.03**
Work training or apprentice, elementary occupations and other	0.83 (0.51, 1.37)	0.48

Work ability score (WAS) as dependent variable, dichotomized into low/moderate and good/excellent. CI: confidence interval. OR: Odds ratio. EQ-5D: EuroQol 5 dimensions. VAS: Visual analogue scale (100 = best). OR adjusted for age, gender, region, diagnosis, comorbidities and baseline work ability. ^a^ Reference group. Bold indicates *p* < 0.05. Pseudo R^2^ = 0.22.

## Appendix B

**Table A3:** List of diagnoses comprising the “other” category.

Diagnosis in the “Other” Category (n = 927)	n (%)
Cardiological diseases	56 (6.04)
Lifestyle-related illnesses	284 (30.63)
Neurology	274 (29.56)
Diseases in the ear	124 (13.38)
Mental illnesses	69 (7.44)
Other *	120 (12.94)

* Other was already a category that could be chosen when reporting diagnoses.
